# Physically Challenging Song Traits, Male Quality, and Reproductive Success in House Wrens

**DOI:** 10.1371/journal.pone.0059208

**Published:** 2013-03-19

**Authors:** Emily R. A. Cramer

**Affiliations:** 1 Department of Neurobiology and Behavior, Cornell University, Ithaca, New York, United States of America; 2 Cornell Lab of Ornithology, Ithaca, New York, United States of America; 3 Natural History Museum, University of Oslo, Oslo, Norway; CNRS, Université de Bourgogne, France

## Abstract

Physically challenging signals are likely to honestly indicate signaler quality. In trilled bird song two physically challenging parameters are vocal deviation (the speed of sound frequency modulation) and trill consistency (how precisely syllables are repeated). As predicted, in several species, they correlate with male quality, are preferred by females, and/or function in male-male signaling. Species may experience different selective pressures on their songs, however; for instance, there may be opposing selection between song complexity and song performance difficulty, such that in species where song complexity is strongly selected, there may not be strong selection on performance-based traits. I tested whether vocal deviation and trill consistency are signals of male quality in house wrens (*Troglodytes aedon*), a species with complex song structure. Males’ singing ability did not correlate with male quality, except that older males sang with higher trill consistency, and males with more consistent trills responded more aggressively to playback (although a previous study found no effect of stimulus trill consistency on males’ responses to playback). Males singing more challenging songs did not gain in polygyny, extra-pair paternity, or annual reproductive success. Moreover, none of the standard male quality measures I investigated correlated with mating or reproductive success. I conclude that vocal deviation and trill consistency do not signal male quality in this species.

## Introduction

In species with traditional sex roles, intrasexual selection favors male traits that enhance their ability to out-compete other males for mating opportunities, and intersexual selection favors traits that make males more attractive to females [1]. Sexual signals are generally thought to be honest signals of male quality, because signal receivers should rapidly evolve to disregard dishonest signals [2], [3]. For a signal to honestly indicate male quality, there must be a cost of or constraint on signal production that makes it expensive or impossible for low quality males to produce high quality signals [4], [5] (reviewed in [2], [3]). Signals that incorporate challenging motor displays may be particularly likely to be costly or constrained, and therefore to be honest signals [6]. Signal complexity may also be under strong selection (e.g., [7]), and there may be divergent selective pressures such that species selected to have more complex songs are not under selection for performance-based signals, while species with strong selection on performance-based signals may not be under strong selection for signal complexity [8].

Birds’ songs are elaborate signals that probably represent a substantial motor challenge because they involve coordinating movements of the respiratory system, the vocal organ (the syrinx), and the upper vocal tract [9], [10]. As such, they have been extensively studied with regard to honest signaling [7], [11]. Vocal deviation and consistency are two aspects of song that have recently received a great deal of attention as potential honest signals of male quality, because they appear to represent particularly challenging motor displays.

Vocal deviation is a measure of how quickly the bird modifies sound frequency in a trill, or a series of repeated syllables [12], [13]. In trill production, a bird cannot simultaneously maximize frequency bandwidth and trill rate [12]: a broad frequency bandwidth requires a large-magnitude change in the volume of the oropharyngeal cavity [10] and in beak gape [14], while a high trill rate requires rapid repetition of those changes. Due to mechanical constraints, then, there is an upper limit on the combination of frequency bandwidth and trill rate [12]. Deviation from this performance limit is thought to reflect trill difficulty: “low deviation” trills that combine a relatively broad frequency bandwidth with a relatively fast trill syllable repetition rate represent a greater physical challenge [12]. In several species, females prefer males with lower deviation, more challenging trills [15]–[20], and may even alter investment in eggs depending on the vocal deviation of males’ songs ([21] and references therein). Vocal deviation correlates with male quality (e.g., age and mass, [22], [23] but see [24]) and affects how males respond to playback in several species [25]–[29] but see [30]).

Consistency is a measure of how precisely a sound is reproduced each time the bird repeats it, and it can be measured at the level of either whole songs or individual, repeated syllables. Producing consistent songs and trills might require an especially high degree of integration across multiple brain regions, including the direct motor control of respiratory, syringeal, and vocal tract muscles [9], [31]–[33]. Complicating this putative honesty mechanism, the anterior forebrain pathway of song learning actively introduces variability (i.e., reduces song consistency) under many conditions, suggesting that males do not sing at their maximum song consistency at all times (reviewed in [33]). Though the mechanism of signal honesty is not fully elucidated, a growing body of literature supports the hypothesis that consistency is an important signal of male quality in birds: consistency is positively associated with field indicators of female preference [20], [32], [34] and male quality [34]–[37], and trills with different consistencies elicit different responses from males in playbacks [36], [38] (but see [30]). Consistency in the timing of notes within a song can be negatively affected by experimentally-induced stressful rearing conditions in zebra finches (*Taeniopygia guttata*) [39].

Consistency and vocal deviation have attracted substantial attention, and to date most evidence suggests that they carry honest information about male quality. However, behavioral ecologists recognize that other aspects of song can also affect the difficulty of song production [40], [41] and that sexual selection can favor different traits in different taxa (e.g., [8]). Further study is therefore needed, especially in species with complex song structure, to determine how widely vocal deviation and trill consistency are used as signals. I studied whether vocal deviation and trill consistency affect male mating success in the house wren (*Troglodytes aedon*). In this species, song is the most probable target of sexual selection, as the species is dull-colored and males are only slightly larger than females [42], but males sing much more frequently and with more elaborate song structure than females do [43], [44]. Males sing at a high rate during territory establishment and mate attraction [45], and females are more likely to visit a nest box if male song is broadcast from it [46], though they may chose mates based on territory characteristics rather than male or song characteristics [47].

In this study, I tested the hypotheses that vocal deviation and trill consistency are honest indicators of male quality and that they affect mating and reproductive success in house wrens. Neither the influence of fine-scale acoustic song features on female choice, nor the relative importance of female choice and male-male competition in determining reproductive success, is known in this species. Variation in male reproductive success depends heavily on success in attracting a secondary (i.e., polygynous) female, and to a lesser extent, on success in extra-pair (EP) paternity [48], which accounts for about 15% of offspring [49]. Both polygyny and EP paternity can be affected by female preferences and by male competitive ability (e.g., [50], [51]), so I discuss success in polygyny and EP paternity as variation in “mating success” rather than in terms of attractiveness. A direct test of female preference in this species is not feasible, as females do not respond well to captivity (pers. obs.) However, male house wrens do not respond differently to songs that differ in vocal deviation and trill consistency [30].

After investigating how vocal deviation and trill consistency related to each other, I tested the following predictions of the hypotheses that vocal deviation and trill consistency reflect male quality and affect mating and reproductive success. 1) Singing ability and male phenotypic quality should be correlated. Specifically, male quality measures should negatively correlate with vocal deviation (since lower vocal deviation indicates a more challenging trill) and positively correlate with trill consistency. 2) Singing ability should relate to mating success. Polygynous males, males that sire EP offspring in other broods, and males that maintain a high proportion of within-pair (WP) paternity within their own broods should sing with lower vocal deviation and higher trill consistency. 3) Males with higher annual reproductive success (i.e., total number of offspring sired) should sing with lower vocal deviation and higher trill consistency. I further tested for relationships among quality measures and mating and reproductive success, for completeness.

## Methods

### Ethics Statement

All protocols were approved by Cornell University’s Institutional Animal Care and Use Committee (Protocol 2007-0123), and appropriate state and federal permits were obtained (Federal Bird Banding subpermittee under Sandra Vehrencamp, 20954; New York State License to Collect or Possess, 1231).

### Field Procedures

I studied house wrens nesting in boxes at two partially-wooded sites at the Cornell University Research Ponds in Ithaca, NY (see [52], [53] for details on study sites). I captured, banded, and bled most breeding adults and offspring between April and August 2008–2011. For adults, I measured wing chord (Avinet wing rule, 0.5 mm accuracy), tarsus length (SPI Dial calipers, 0.1 mm accuracy), and weight (to the nearest 0.1 g with a Pesola spring scale). I monitored all breeding attempts on the field sites and banded chicks at approximately 7 days of age. To prevent premature fledging, I did not continue to count offspring after banding; in estimating reproductive success, I assumed that all banded chicks fledged unless I saw obvious signs of depredation or nestling starvation. Annual reproductive success for each male was the sum of the number of chicks fledged from all his nests, accounting for gains or losses due to EP paternity. Some nests were involved in brood size manipulations, and these males were not included in analyses of total reproductive success.

### Trill Measurements

House wren songs typically begin with relatively low-amplitude introductory notes and end with a series of trills, with each trill composed of a different syllable type. Within each trill, mean pitch is generally fairly constant, but each succeeding trill usually occurs at a lower mean pitch than the one before [30].

trill measurements, I used recordings from playbacks conducted in 2009 and 2010 (see details in [30]), made with a Marantz PMD 690 recorder and Sennheiser ME 67 or MKH 816 shotgun microphone at a 48 kHz sampling rate and a 16-bit depth. I isolated individual songs from each playback recording in Syrinx PC [54]. I measured frequency bandwidth (the bandwidth encompassing 99% of the sound energy in the syllable) and trill rate (syllables/sec) for each trill, using spectrograms in RavenPro 1.4 [55] (Hann window with 80.1% overlap in the time domain, giving 111 sample hop size, 4096 DFT size and 11.7 Hz grid). Though measurements were visualized on the spectrogram, they were calculated from the power spectrum and so should be robust to variation in amplitude due to differences in the distance between the recordist and the bird. I performed upper-bound regression on the relationship between frequency bandwidth and trill rate to estimate the performance limit on frequency modulation [12] and found the predicted triangular distribution [30]. Vocal deviation was the orthogonal distance from each trill to this performance limit (estimated limit: frequency bandwidth = −168.50* trill rate (in Hz) +6019 Hz).

For trill consistency, I cross-correlated the spectrograms of individual syllables within trills using SoundXT [56] with the following settings: FFT length 1024; data length 50%; Hann window; 80% overlap; masking method broadband; 50% masking; masking adjustment bias; spec pairwise; correlator type matrix standard method. I bounded the cross correlation at 200 Hz above the highest high frequency and 200 Hz below the lowest low frequency for the trill to minimize interference from background noise. Trill consistency was the mean cross correlation score within a single trill. I did not allow the cross correlator to shift sounds in frequency, which would have allowed a comparison of note “shape” regardless of pitch, because the signaling value of pitch changes within a trill is unknown. Without an a priori expectation that either total similarity (including similarity of pitch) or shape similarity (eliminating pitch differences) is the biologically relevant signal, I decided to cross-correlate notes at their actual pitches for comparability with other studies on consistency.

I measured four acoustic covariates of vocal deviation and trill consistency: pitch, trill duration, timing of the trill within the song, and trill type. I defined pitch as the mean high frequency of the trill [30]. The timing within the song was the time from the beginning of the song to the beginning of the trill. All trills used in this study could be assigned to one of eight syllable types that are shared among males in the population (approximately 96% of trills can be assigned to one of these eight types [30]), and 5.88±0.18 (mean ± SE) syllable types were included per male per year (range 2–8).

The total sample size was 4569 trills (mean ± SE, range: 62.6±4.14, 8–193 trills per male per year, distributed across 59 males, with 14 males measured in two years).The unit of analysis in this study was the trill; individual songs contributed 2.11±0.02 measureable trills to the study (range, 1–6). Five hundred thirty-eight trills were recorded before playback, and the remaining 4031 were recorded during or immediately after playback. Vocal deviation does not differ between the pre-playback and the during/post-playback time periods, while trill consistency increases slightly but significantly from pre-playback to during/post playback (unpublished results). To maintain high statistical power, I included all trills in the analyses. Results were qualitatively unchanged if I instead restricted analyses to trills recorded during/post playback, which should equalize motivational state across males and allow for a more accurate between-male comparison.

### Male Phenotypic Quality

I captured 125 males a total of 253 times over four breeding seasons, at varying stages of nesting. I measured the following putative male quality attributes: size, body condition, age, health, and aggressiveness. I tested for correlations between measures of male quality and trill quality, mating success, and reproductive success from the same year only. Two males banded in a previous year were recorded in 2009 or 2010 but not captured, and therefore are not included in male quality correlations. Of the remaining 71 male-years for recording, 45 males were captured on the same day as song recording, and 26 were recorded 26±3.6 days before capture. A subset of males and quality measurements from 2009–2011 are also included in Cramer et al. [57], addressing other questions.

I estimated body size as wing chord, tail length, and tarsus length; for males measured multiple times in a year, I used the mean of the measurements from the year. I could not collapse these variables using principal components analysis because it is statistically inappropriate to include multiple captures for only a subset of individuals, but wing and tail measures increased with age, so it was necessary to use measurements from the appropriate year for males captured in multiple years. For body condition, I used the standardized residual of a regression of weight on tarsus, controlling for date and time of day captured. In correlations with trill quality, I used the body condition score closer to the recording date for males captured multiple times in a year. For correlations with mating and reproductive success, I used the first measure of body condition from that year, though results were unchanged if I instead randomly chose a measurement occasion (not shown). Measurement repeatability was highly statistically significant (sensu [58], r>0.68, F>5.18, p<0.0001 for tarsus, wing, tail, and weight, n = 112–114 measurements on 50–51 males for tarsus, tail, and weight; n = 62 measures and 28 males for wing, for captures within the same year).

I could assign age only for a subset of individuals (84 male-years) that had been banded on-site in a previous year. I categorized males as second-year (SY) if they had been banded as nestlings the previous year (i.e., this was their first breeding season) and after-second-year (ASY) if they had been banded as adults in a previous season. I did not make finer-scale age assignments among ASY males that were present multiple years.

In 2009, I used two ecoimmunology techniques to assess male health (see [57] for details). Briefly, I followed procedures in [59] to measure the bactericidal capacity of 10-µl whole blood samples collected from the brachial vein after ethanol sterilization. Scores for the bactericidal assay are thought to increase with improved innate immunity [59]. All samples for this data set were collected during pre-nestling breeding stages. I also took blood smears and had the ratio of heterophiles:lymphocytes counted by the Animal Health Diagnostic Center at Cornell University Veterinary College; the heterophile:lymphocyte ratio increases in response to stress [60]. For males that had two blood smears taken, I used the one closer to the recording date for song analyses, and I used the first measure for analyses with mating and reproductive success. Results are unchanged if I instead randomly chose which measure to include in the latter analyses (not shown).

I derived aggression scores from playback experiments conducted in 2008, 2009, and 2010 as part of other studies [30], [61]. Briefly, for each playback experiment, I did a series of presentations to each male, with “song-bouts” during which a single stimulus song was repeated consecutively at a biologically relevant song rate. Song-bouts were separated by periods of silence. In 2008, a single song stimulus was repeated for six song-bouts [61]. In 2009 and 2010, each male heard three song-bouts, with each song-bout repeating a different manipulation of a single song. I found no evidence that the stimulus manipulation affected male response [30]. Each year’s experiment had other unique attributes (e.g., speaker brand and distance to the nest box) that could affect responses to playback, so I included a year/experiment variable in analyses. For all playback experiments, I calculated the mean song rate during the entire playback trial. I calculated the mean proportion of time the male spent within 5 m of the speaker and the mean number of flights across the speaker (i.e., within a 2 m ring across the speaker) during song-bouts only, since there were many zero values during silent periods. No measure of aggressiveness was repeatable across years (sensu [62], controlling for experimental protocol, all r<0.3, all p>0.9, calculated with 20 males exposed to 2 or 3 playback experiments each, and a total of 43 playbacks). Each male was the subject of only one playback experiment per year, and each male heard a unique stimulus set.

### Paternity Analysis, Male Mating Success, and Reproductive Success

I followed the PCR protocol of [53] and genotyped all adults as well as 857 offspring from 182 nests using a panel of 7 microsatellite markers. Because of financial constraints, no genotyping was conducted for one study site for 2008. I conducted paternity analysis using Cervus 3.0, including the social mother as a known parent [63]. I confirmed mis-matching alleles by re-genotyping both the parent and the offspring. To most conservatively estimate EP paternity, I attributed a chick to EP paternity if it had more than one trio-wise mismatch with its social parents that could not be attributed to a null allele. In assigning EP sires, I allowed EP fathers to have a single null-allele mismatch [64] with his putative offspring. Nests from 2008 were also included in [53].

I defined WP success as the proportion of social offspring that a male sired, calculated separately for each year but combining all social nests within a year. Results were unchanged if I weighted analyses by the number of social offspring with paternity data (not shown). A male was considered to have EP success if he was an EP sire of at least one chick in that year. Similar results were found if I instead analyzed the total number of EP offspring sired in a year (not shown). For EP success analyses, I excluded males that deserted the study site immediately after capture. Because there was suitable habitat for house wrens surrounding my study site, I may have failed to detect EP offspring of males that bred on site but that only gained EP success off-site. Failed detections should not bias the results, as they should be random with respect to the song variables measured.

I considered a male polygynous if he attracted a female to a second nest box while his primary female still had an active nest (i.e., simultaneous polygyny, as in [65]; results remain qualitatively unchanged if I also consider males polygynous if they had different females for each brood). For three nests, I was unable to determine whether the male was simultaneously polygynous, and these nests were excluded. For ease of discussion, I consider males to have higher “mating success” if they were polygynous, sired a high proportion of their social offspring, and/or gained EP success elsewhere. In paired comparisons of EP males and the WP males they cuckolded, the EP males are considered to have higher mating success.

For an additional four nests, I could not distinguish rapid mate-switching from complete loss of WP success, due to a gap in field observations, and I excluded these males from analyses of WP success. Two additional males were excluded from analyses of WP and reproductive success because a majority of the offspring had two mismatches that could be attributed to null alleles. While having two null alleles is relatively unlikely, it is possible given the null allele rates in the population (Table 1), so the genetic sire is unclear. For these six nests, where genetic fathers could be assigned with high confidence, chicks were included towards males’ total genetic reproductive success. Reproductive success was defined as the number of chicks a male sired (WP and EP) that fledged.

**Table 1:** Descriptive statistics for microsatellite loci used in paternity analysis.

Locus	N alleles	Observed heterozygosity	Expected heterozygosity	Null allele frequency
PCA^1^	12	0.533	0.519	−0.021
TA-A5-15^2^	10	0.596	0.680	0.059
TA-A5-2^2^	18	0.799	0.893	0.054
TA-B4-2^2^	17	0.847	0.837	−0.008
TA-C3(B)-2^2^	28	0.900	0.899	−0.001
TA-C6-7^2^	7	0.598	0.679	0.063
ThP114^3^	17	0.882	0.885	0.001

Data are from 229 adult birds sampled over 4 years (TA-A5-15 did not amplify in one individual). Statistical analysis was conducted in Cervus 3.0.

1 Dawson et al. 2000 [89].

2 Cabe and Marshall 2001 [90].

3 Brar et al. 2007 [91].

### Statistical Analysis

I first tested for associations among different measures of mating success using logistic regression with year as a fixed effect and male identity as a random effect. To test whether the likelihood of losing WP paternity depended on a nest’s polygyny status (i.e., whether that nest belonged to a monogamous male, or was the primary versus secondary nest of a polygynous male), I coded each nest as containing all WP or at least one EP offspring, and also as belonging to one of these three polygyny statuses.

To determine how much variation in trill measures was between-male versus within-male, I assessed the proportion of variation in vocal deviation and trill consistency that was attributable to a random effect of male identity, in a model including fixed effects of year and four acoustic covariates: trill type, pitch, the time of the trill within the song, and trill duration (repeatability, sensu [62]). Multicollinearity among acoustic covariates was not problematic, as the variance inflation factors from models without the random effect were all less than 5. To test whether vocal deviation and trill consistency were correlated, I followed the methods of [66]: I first calculated the mean consistency for each male - trill type - year combination separately, and I then calculated the difference from each trill to the mean for that male - trill type - year combination. I used both of these variables together in a model including the four acoustic covariates (trill type, pitch, the time of the trill within the song, and trill duration) and year as fixed effects and male identity as a random effect, to predict vocal deviation as a function of between- and within-male variation in consistency [66]. The between-male effect was estimated using the mean for the male - trill type - year combination term, and the within-male effect was estimated using the difference from each trill to this mean.

I assessed whether vocal deviation and trill consistency were associated with male quality measures by fitting general linear mixed models with the song measure as the response variable, fixed effects of year, a single male quality measure, the four acoustic covariates (above), and a random effect of male identity. Because the heterophile:lymphocyte ratio and the bactericidal assay were analyzed in a single year, those models did not include year effects.

Next, I assessed whether any of the male or trill quality measures was related to mating success. I constructed a separate general linear mixed model for each measure of mating success (polygyny, WP success, and EP success) and for each male quality or trill measure. Data were missing from different variables for different males, so constructing separate models allowed me to maximize sample size for each analysis. Each model used the song or male quality measure as a response variable, and the following predictors: a measure of mating success, a fixed effect of year (except for health measures), and a random effect of male identity (except for health measures). For analyses of trill measures, I also included the four acoustic covariates (above). To test for relationships between reproductive success and trill quality, I used the trill quality measure as the response variable, with reproductive success, year, and the four acoustic covariates as fixed effects and male identity as a random effect. This approach reverses the logical response and independent variables, but the reversal is necessary to account for the non-independence of trill measurements (i.e., multiple trills were measured per male, and the random effect of male identity can only control for this pseudoreplication if trill measures are the response variable), and it allows me to control for the acoustic covariates. Moreover, the goal of the analysis is to measure the association between the two variables, and the strength of the association should be unaffected by which variable is response versus independent.

For analyzing the relationship between reproductive success and male quality and mating success, I used reproductive success as the response variable, with fixed effects of year and the male quality/mating success variable of interest, and male identity as a random effect.

For paired comparisons of EP males to the WP males they cuckolded, I conducted paired t-tests for each male quality measure. For trill measures, because I had many measurements for each male, I constructed general linear mixed models to predict vocal deviation or trill consistency, with role (EP versus WP), year, and the four acoustic covariates as fixed effects, and random effects of male identity and a grouping variable to associate EP males with the WP males they cuckolded.

All analyses used response and independent variables measured in the same year (e.g., trills measured in 2009 were compared to male quality measures, mating success, and reproductive success in 2009 only). Vocal deviation, size measures, body condition, and song rate in response to playback approached normal distributions and were not improved by transformation. Trill consistency was transformed as −log(1-trill consistency), and I took the square root of reproductive success plus one. The ratio of heterophiles:lymphocytes was log-transformed, flights across the speaker were raised to the power of 0.55, the proportion of time within 5 m of the speaker was arc-sine square-root transformed. Following [67], variables were not transformed when they were used as predictors. Transformation was unnecessary for paired tests of EP males and the WP males they cuckolded, as the differences were normal. The percent bacteria killed was strongly skewed and could not be transformed for normality.

Most tests were performed in JMP 7.0 [68], which uses the Kenwood-Roger approximation for degrees of freedom in mixed models. Degrees of freedom are therefore intermediate between the number of individuals and the total number of measurement events. Mixed models with a categorical response variable (e.g., whether male age differed between different levels of mating success) were performed in R version 2.15.1 [69] using the LMER function with a binomial error distribution [70]. The statistical significance of the repeatability of song and aggression measures was determined using the package nlme [71] following [67]. Where necessary, I used the Wald test in package AOD [72] to find significance of a factor with multiple levels.

To correct for multiple testing, I used false discovery rate [73], implemented in R. I conducted table-wise corrections (with tests of mating success combined across tables). P-values listed in the tables are un-corrected, and I note whenever statistical significance changed after correction for multiple testing. I calculated standardized effect sizes and their confidence intervals according to [74], and for mixed effects models, using R code from [75] for non-central confidence intervals. Following [74], I consider effect sizes small, medium, or large with r = 0.1, 0.3, or 0.5, or d = 0.2, 0.5, or 0.8, respectively.

## Results

### Paternity

The seven microsatellite markers gave high power for determining paternity (non-exclusion probability for the parental pair, 0.0003%, estimated using all adult genotypes). Several markers had low levels of null alleles (Table 1): single pair-wise mismatches consistent with being null alleles occurred in over 40 chicks for each parental sex. Sixteen of 857 offspring had single mismatches with their social mothers that could not be attributed to null alleles, and nine offspring had single non-null mismatches with their social fathers. Non-null mismatches between the mother and offspring may be due to mutation, since intra-specific brood parasitism has not been reported in this species [65]. I therefore allowed these single mismatches with putative parents of either sex. Across all years, 13.5% (116/857) of offspring in 37.6% (68/181) of nests were EP young. Results presented here are not changed substantially if these offspring with single non-null mismatches were instead attributed to EP paternity.

EP and WP success were not related: 39.7% (27/68) of males with complete WP success gained EP success, compared to 35.6% (21/59) of males that lost at least some WP success (n = 127 male-years, 96 males, effect of EP sire status, z = −0.79, p = 0.43). WP success and polygyny were also unrelated (c.f. [65]). For monogamous males, 36.7% (44/120) of nests contained at least one EP offspring; 44.8% (13/29) of polygynous males’ primary nests and 31.0% (9/29) of polygynous males’ secondary nests contained EP offspring (n = 97 males, 178 nests, χ^2^_2_ = 2.3, p = 0.32). Polygyny also did not relate to success gaining EP offspring elsewhere: 33.3% (39/117) of monogamous males and 48.4% (15/31) of polygynous males gained EP success (z = 1.51, p = 0.13, 109 males and 148 observations).

### Acoustic Covariates and Repeatability of Trill Measures

In most models, the four acoustic covariates significantly affected vocal deviation and trill consistency measurements (not shown). Vocal deviation was generally lower (i.e., higher performance trills) when the trill was higher-pitched (also see [30]), had a longer duration, and occurred later in the song. Trill consistency generally increased (i.e., higher performance trills) when the trill was lower-pitched, shorter, and later in the song. Syllable types differed consistently in both vocal deviation and trill consistency.

Vocal deviation and trill consistency were weakly but significantly repeatable: 17.6% of the variation in vocal deviation and 29.5% of the variation trill consistency was attributable to a random effect of male identity (controlling for trill type, pitch, time in the song, and trill duration, p<0.0001; sensu [62]). Performance was significantly, positively related between the two measures: trills with lower vocal deviation (i.e., higher performance) had higher consistency due to both between-male effects (effect estimate ± SE, −5.55±0.44, t = −12.50, p<0.0001) and within-male effects (−3.02±0.29, t = −10.57, p<0.0001). The effect size of these relationships was small to medium, with the partial r (95% confidence interval) for the between-male effect being −0.16 (−0.19, −0.14) and for the within-male effect being −0.14 (−0.16, −0.12).

### Correlations between Trill Quality and Male Quality

Few correlations between trill quality and male quality were statistically significant (Table 2). Vocal deviation correlated with tail length, such that males with longer tails sang lower-deviation (i.e., more challenging) trills (Table 2), though these results were not robust to correction for multiple testing. Trill consistency correlated with age, wing chord, and response to playback, and these relationships remained statistically significant following correction for multiple testing (Table 2). The effect size for age was medium to large, while the other effect sizes were small.

**Table 2:** Male quality measures in relation to vocal deviation and trill consistency, with effects that remained significant after correction for multiple testing in bold.

Male quality measure	N males (trills)	Trill measurement	Effect estimate ± SE	Partial r or Cohen’s d (95% CI)	T_df_ (p)
Tarsus	58 (4439)	Voc Dev	0.203±0.158	0.017 (−0.009, 0.044)	t_94.7_ = 1.28 (0.20)
		Consistency	0.041±0.056	0.009 (−0.016, 0.034)	t_136.9_ = 0.73 (0.46)
Wing	58 (4439)	Voc Dev	−0.001±0.039	0.000 (−0.027, 0.026)	t_241_ = −0.02 (0.99)
		**Consistency**	**0.082±0.013**	**0.082 (0.057, 0.107)**	**t_497_ = 6.55 (0.0001)**
Tail	58 (4439)	**Voc Dev**	**−0.107±0.041**	**−0.035 (−0.062, −0.008)**	**t_79.7_ = −2.61 (0.01)**
		Consistency	0.021±0.014	0.019 (−0.006, 0.044)	t_108.4_ = 1.49 (0.14)
Condition	58 (4439)	Voc Dev	0.073±0.124	0.008 (−0.019, 0.035)	t_118_ = 0.59 (0.56)
		Consistency	−0.037±0.043	−0.011 (−0.036, 0.014)	t_189.3_ = −0.88 (0.38)
Age	34 (2659)	Voc Dev	0.003±0.071	0.006 (−0.272, 0.283)	t_520.2_ = 0.04 (0.97)
		**Consistency**	**0.090±0.020**	**0.637 (0.353, 0.920)**	**t_467.2_ = 4.45 (0.0001)**
H:L	43 (2746)	Voc Dev	−0.067±0.103	−0.011 (−0.046, 0.023)	t_40.3_ = −0.66 (0.52)
		Consistency	0.025±0.040	0.010 (−0.021, 0.042)	t_38.1_ = 0.64 (0.53)
Bact Cap	42 (2682)	Voc Dev	0.062±0.307	0.004 (−0.031, 0.038)	t_40.9_ = 0.20 (0.84)
		Consistency	−0.063±0.111	−0.009 (−0.042, 0.023)	t_38.2_ = −0.56 (0.58)
Song Rate	58 (4530)	Voc Dev	0.001±0.016	0.001 (−0.026, 0.027)	t_752.2_ = 0.06 (0.95)
		**Consistency**	**0.035±0.005**	**0.094 (0.069, 0.119)**	**t_1350_ = 7.37 (0.0001)**
Flight Rate	58 (4530)	Voc Dev	0.000±0.029	0.000 (−0.026, 0.027)	t_333.9_ = 0.01 (0.99)
		Consistency	0.010±0.009	0.014 (−0.011, 0.039)	t_659.7_ = 1.10 (0.27)
Time Close	58 (4530)	Voc Dev	0.182±0.121	0.020 (−0.006, 0.047)	t_1028_ = 1.50 (0.13)
		**Consistency**	**0.127±0.036**	**0.044 (0.019, 0.068)**	**t_2119_ = 3.52 (0.001)**

Trill measures were the dependent variables, and male quality measures were the independent variables, in mixed-effects models controlling for male identity, year, trill type, pitch, trill duration, and the time of the trill in the song. For age, the effect estimate is the difference between second-year and after-second-year males, and the effect size is Cohen’s d. For continuous variables, effect estimates are the slope, and the effect size is partial r. Song rate, flight rate, and time close are responses to playback. Abbreviations : Voc Dev: Vocal Deviation. H:L, Heterophile:Lymphocyte ratio. Bact Cap, Bactericidal Capacity. CI, Confidence Interval.

In the cross-sectional analysis, after-second year (ASY) males sang more consistently than second-year (SY) males (Table 2; back-transformed least squares means ± SE, ASY 0.83±0.03, 31 males; SY 0.79±0.04, five males). Sample sizes for longitudinal analysis were very limited, but agreed with this pattern: two males recorded as SY in 2009 and ASY in 2010 increased their trill consistency with age (SY 0.77±0.04; ASY 0.80±0.04; F_1,275_ = 11.87, p = 0.0007, n = 288 trills, restricted to syllable types recorded in both years). In contrast, four males were recorded as ASY in both 2009 and 2010, and their trill consistency decreased with age (2009 0.85±0.08; 2010 0.83±0.08; F_1,559.4_ = 8.53, p = 0.004, n = 571 trills, restricted to syllable types recorded in both years).

The apparent effect of wing chord on trill consistency is likely driven by age effects on both size and consistency: older males sing more consistently and had longer wings than SY males (ASY wing chord 51.2±0.17 mm, SY 49.7±0.36, F_1,63.33_ = 15.63, p = 0.0002, n = 80 observations). When I simultaneously assessed the effect of age and wing chord on consistency, the age effect remained highly significant while wing chord did not (not shown). Age effects did not appear to drive the relationship between tail length and vocal deviation.

Most measures of male quality were not strongly inter-correlated in simple regressions, with the following exceptions. All three playback response measures were positively correlated (song rate with flights across the speaker: r^2^ = 0.11, p = 0.001; song rate with proportion time close to the speaker: r^2^ = 0.06, p = 0.01; flights across the speaker with time close to the speaker, r^2^ = 0.36, p<0.0001). These three variables were not collapsed with principal components analysis because they showed different patterns in analyses [27]. Wing chord correlated positively with body condition, tarsus, and tail. The correlation between tarsus and tail only approached significance (p = 0.07). Age only affected wing and tail measures (see above).

### Trill and Male Quality: Relation to Male Mating Success

Trill quality did not correlate with polygyny (Table 3). Males that lost a higher proportion of WP success had lower vocal deviation and higher trill consistency–both putatively “better” song characteristics (Table 4). Similarly, males that did not gain EP success in other nests on site had lower vocal deviation than males that gained EP success, although the males that gained EP success did have higher trill consistency (Table 5). Despite the statistical significance of these patterns at the population level, paired comparisons of EP males to the WP males they cuckolded revealed no differences in trill quality, even with high sample size (Table 6).

**Table 3:** Polygyny in relation to trill and male quality; no effects remained significant after correcting for multiple testing.

Trill or male qualitymeasure	N males (obs.)	Estimated difference ± SE	Cohen’s d or partial r (95% CI)	Test statistic_df_ (p)
Vocal Deviation	50 (3897)	0.026±0.036	0.041 (−0.070, 0.152)	t_1386_ = 0.73 (0.47)
Consistency	50 (3897)	−0.006±0.01	−0.033 (−0.137, 0.072)	t_2293_ = −0.61 (0.54)
Tarsus	120 (164)	0.024±0.021	0.275 (−0.198, 0.746)	t_49.93_ = 1.15 (0.25)
Wing	120 (164)	0.116±0.112	0.440 (−0.393, 1.270)	t_76.23_ = 1.04 (0.30)
Tail	120 (163)	0.054±0.092	0.161 (−0.375, 0.695)	t_50.55_ = 0.59 (0.56)
Condition	120 (164)	−0.032±0.039	−0.335 (−1.156, 0.488)	t_89.87_ = −0.80 (0.42)
Age	56 (79)	1.421±2.894	0.086 (−1.544, 1.716)	z = 0.49 (0.62)
H:L	39 (39)	−0.009±0.145	0.022 (−0.623, 1.375)	t_37_ = −0.06 (0.95)
Bactericidal assay	39 (39)	0.026±0.047	−0.201 (−0.848, 1.711)	t_37_ = 0.57 (0.58)
Song Rate to PB	64 (85)	0.127±0.323	0.223 (−0.898, 1.342)	t_75.54_ = 0.39 (0.70)
Flights in PB	64 (85)	0.175±0.092	1.068 (−0.049, 2.180)	t_81_ = 1.90 (0.06)
Time Close in PB	64 (85)	0.079±0.051	0.897 (−0.250, 2.038)	t_69.71_ = 1.55 (0.13)

Trill and male quality measures were the dependent variables, and polygyny status (monogamous vs. polygynous) was the independent variable in mixed-effects models controlling for male identity, year, and (for trill measures only) trill type, pitch, trill duration, and the time of the trill in the song. Age, the only categorical dependent variable, was modeled with logistic regression; the estimated difference is the difference in log-odds of being an after-second-year male if the male is polygyous, and the effect size is partial r. For continuous variables, estimated differences are between successful and unsuccessful males, with a positive difference indicating that the more successful males had a higher score for quality, and the effect estimate is Cohen’s d.

Abbreviations: obs., observations. CI, confidence interval. H:L, Heterophile:Lymphocyte ratio. PB, playback.

**Table 4:** Within-pair paternity success relative to trill and male quality, with effects that remained significant after correcting for multiple testing in bold.

Trill or male quality measure	N males (obs.)	Effect estimate ± SE	Partial r (95% CI)	Test statistic_df_ (p)
**Vocal Deviation**	**43 (3354)**	**1.202±0.284**	**0.067 (0.035, 0.098)**	**t_266.9_ = 4.24 (0.0001)**
**Consistency**	**43 (3354)**	**−0.300±0.087**	**−0.051 (−0.081, −0.022)**	**t_518.4_ = −3.44 (0.001)**
Tarsus	94 (124)	0.167±0.143	0.060 (−0.041, 0.158)	t_52.7_ = 1.17 (0.25)
Wing	94 (124)	−0.126±0.567	−0.016 (−0.159, 0.128)	t_79.8_ = −0.22 (0.82)
Tail	94 (124)	−0.310±0.594	−0.030 (−0.14, 0.082)	t_63_ = −0.52 (0.60)
Condition	94 (124)	−0.161±0.214	−0.066 (−0.232, 0.106)	t_113.5_ = −0.75 (0.45)
Age	45 (62)	−0.985±21.622	−0.011 (−1.598, 1.576)	z = −0.05 (0.96)
H:L	32 (32)	−0.579±0.764	−0.137 (−0.445, 0.217)	t_30_ = −0.76 (0.45)
Bactericidal assay	32 (32)	−0.112±0.229	−0.089 (−0.408, 0.261)	t_30_ = −0.49 (0.63)
Song Rate to PB	53 (68)	−1.041±1.811	−0.073 (−0.309, 0.176)	t_62.1_ = −0.57 (0.57)
Flights in PB	53 (68)	−0.982±1.043	−0.117 (−0.341, 0.128)	t_64_ = −0.94 (0.35)
Time Close in PB	53 (68)	−0.114±0.218	−0.067 (−0.305, 0.183)	t_62.5_ = −0.52 (0.60)

Trill and male quality measures were the dependent variables, and the proportion of social offspring sired was the independent variable in mixed-effects models controlling for male identity, year, and (for trill measures only) trill type, pitch, trill duration, and the time of the trill in the song. Age, the only categorical dependent variable, was modeled with logistic regression; the estimated difference is the change in log-odds of being after second year with increasing proportion of social offspring sired. Following the recommendation of [74], the effect size for age is a partial r using within-pair success as a categorical variable (all social offspring sired versus at least on social offspring sired by another male). For continuous variables, effect estimates are the slope, and the effect size is partial r. Abbreviations: obs., observations. CI, confidence interval. H:L, Heterophile:Lymphocyte ratio. PB, playback.

**Table 5:** Extra-pair paternity success relative to trill and male quality, with effects that remained significant after correcting for multiple testing in bold.

Trill or male quality measure	N males (obs.)	Estimated difference ± SE	Cohen’s d or partial r (95% CI)	Test statistic_df_ (p)
**Vocal Deviation**	**49 (3848)**	**0.131±0.036**	**0.184 (0.083, 0.285)**	**t_1291_ = 3.59 (0.001)**
**Consistency**	**49 (3848)**	**0.036±0.011**	**0.164 (0.069, 0.260)**	**t_2095_ = 3.38 (0.001)**
Tarsus	113 (150)	0.009±0.023	0.065 (−0.262, 0.392)	t_54.3_ = 0.39 (0.70)
Wing	113 (150)	0.103±0.107	0.262 (−0.275, 0.797)	t_102.2_ = 0.96 (0.34)
Tail	113 (150)	0.011±0.098	0.020 (−0.333, 0.373)	t_60.7_ = 0.11 (0.91)
Condition	113 (150)	0.024±0.038	0.167 (−0.354, 0.687)	t_106.7_ = 0.63 (0.53)
Age	49 (69)	0.001±10.560	0.041 (−1.412, 1.495)	z = 0.00 (1.00)
H:L	37 (37)	0.048±0.129	0.124 (−1.737, 0.797)	t_36_ = 0.38 (0.71)
Bactericidal assay	38 (38)	0.024±0.039	0.224 (−1.794, 0.889)	t_37_ = 0.62 (0.54)
Song Rate to PB	59 (76)	0.019±0.309	0.026 (−0.822, 0.873)	t_71.9_ = 0.06 (0.95)
Flights in PB	59 (76)	0.303±0.179	0.744 (−0.125, 1.607)	t_71_ = 1.7 (0.09)
Time Close in PB	59 (76)	0.026±0.037	0.310 (−0.549, 1.168)	t_72_ = 0.71 (0.48)

Trill and male quality measures were the dependent variables, and success siring offspring in other males’ nests (sired at least one offspring versus did not sire any) was the independent variable in mixed-effects models controlling for male identity and year, and (for trill measures only) trill type, pitch, trill duration, and the time of the trill in the song. Age, the only categorical dependent variable, was modeled with logistic regression; the estimated difference is the difference in log-odds of being an after-second-year male if the male is polygyous, and the effect size is partial r. For continuous variables, estimated differences are between successful and unsuccessful males, with a positive difference indicating that the more successful males had a higher score for quality, and the effect estimate is Cohen’s d. Abbreviations: obs., observations. CI, confidence interval. H:L, Heterophile:Lymphocyte ratio. PB, playback.

**Table 6:** Paired comparisons of song and male quality measures for extra-pair (EP) males and the within-pair (WP) males they cuckolded; no effect was statistically significant after correcting for multiple testing.

Trill or male trait	N pairs (males)	Mean for WP male	Mean for EP male	Cohen’s d (95% CI)	T_df_ (p)
Vocal Deviation	18 (25)*	8.189	8.218	0.019 (−0.086, 0.123)	t_171_ = 0.35 (0.73)
Consistency	18 (25)*	0.823	0.818	−0.055 (−0.158, 0.049)	t_173.4_ = −1.04 (0.30)
Tarsus	68 (72)	16.84	16.83	0.023 (−0.318, 0.365)	t_67_ = 0.11 (0.91)
Wing	68 (72)	50.70	50.56	0.112 (−0.214, 0.438)	t_67_ = 0.68 (0.50)
Tail	68 (72)	43.5	43.2	0.187 (−0.151, 0.525)	t_67_ = 1.28 (0.20)
Condition	68 (72)	−0.019	0.010	−0.060 (−0.358, 0.239)	t_67_ = −0.39 (0.70)
Heterophile:Lymphocyte	14 (21)	0.694	0.640	0.135 (−0.419, 0.689)	t_13_ = 0.48 (0.64)
Bactericidal assay	15 (23)	0.885	0.872	0.066 (−0.656, 0.788)	t_14_ = 0.18 (0.86)
Song Rate in Playback	28 (31)	7.130	5.897	0.448 (0.006, 0.891)	t_27_ = 2.06 (0.05)
Flights Across Speaker	28 (31)	1.478	1.833	−0.214 (−0.718, 0.290)	t_27_ = 0.84 (0.41)
Time Close to Speaker	28 (31)	0.439	0.426	0.036 (−0.460, 0.531)	t_27_ = −0.14 (0.89)

For song variables, I constructed a mixed model with role (EP vs. WP) as a fixed effect; year, trill type, pitch, trill duration, and timing of the trill within the song as fixed covariates; and male identity and group as random effects. Means for the trill measures are least squares means, and consistency scores are back-transformed. All other tests were paired t-tests. Mean for each group and the effect size (Cohen’s d) for the difference is given.

* 2300 observations were included (for males present in more than one comparison per year, all trills measured for that male in that year were included multiple times).

Male quality did not correlate with mating success (Tables 3, 4, 5) and did not differ between EP and WP males in paired comparisons (Table 6). Before correction for multiple testing, males that flew across the speaker more in response to playback tended to be more likely to be polygynous (Table 3), and WP males sang at a higher song rate in response to playback than the EP males that cuckolded them (Table 6). None of these patterns was robust to correction for multiple testing.

Age effects are of particular interest, as they are important in other house wren populations. However, there were no significant relationships between age and mating success (Table 3, 4, 5). 45.5% (25/55) of ASY birds and 42.9% (3/7) of SY birds were cuckolded: 31.9% (22/69) of ASY and 20.0% (2/10) SY birds were polygynous; and 35.6% (21/59) of ASY and 30.0% (3/10) of SY birds gained EP success (note that these proportions treat males observed in multiple years as independent samples; statistics in Tables 3, 4, and 5 account for non-independence using a random effect of male identity). For paired comparisons of EP and WP sires, I had age data on both males for only 19 pairs; in 16 pairs, both males were the same age, in two pairs, a younger male cuckolded an older male, and in the final pair, the older male cuckolded the younger male.

### Trill Quality, Male Quality, and Male Mating Success: Relation to Annual Reproductive Success

**Figure 1:**
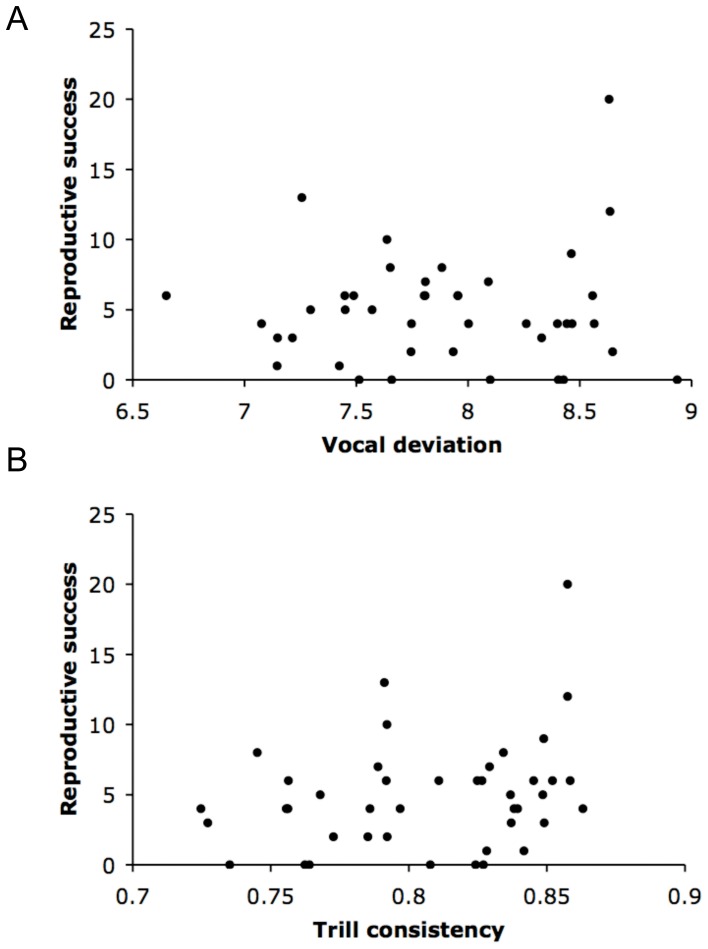
Reproductive success as a function of trill quality. A: Vocal deviation, B: Trill consistency. Trill measures are the least squares means for each male for each year, correcting for trill type, pitch, trill duration, and time in song. Least squares means for trill consistency were back-transformed for visualization. Neither trill measure was significantly correlated with reproductive success in statistical models including all trills measured for each male. For vocal deviation, lower scores correspond to more challenging songs; for trill consistency, higher scores are more challenging songs.

Neither trill consistency nor vocal deviation correlated with reproductive success, though there was a trend with a small effect size for a positive correlation between consistency and reproductive success (Figure 1, Table 7). The three measures of aggression correlated positively with reproductive success, with moderate effect sizes, but these were not significant after correcting for multiple testing (Table 7). Polygyny, WP success, and EP success all had positive and moderate to strong effects on reproductive success, though the effect of EP success was not significant after correction for multiple testing (Table 7).

**Table 7:** Annual reproductive success relative to trill quality, male quality, and male mating success, with the effects that remained statistically significant after correction for multiple testing in bold.

Trill or male trait	N males (obs.)	Effect estimate ± SE	Partial r or Cohen’s d (95% CI)	T_df_ (p)
Vocal Deviation	34 (2227)	0.005±0.015	0.006 (−0.031, 0.044)	t_223.1_ = 0.32 (0.75)
Consistency	34 (2227)	0.009±0.005	0.033 (−0.003, 0.068)	t_444.2_ = 1.82 (0.07)
Tarsus	100 (119)	0.094±0.17	0.054 (−0.137, 0.238)	t_99.1_ = 0.55 (0.58)
Wing	100 (119)	0.036±0.053	0.065 (−0.125, 0.248)	t_113.7_ = 0.67 (0.50)
Tail	100 (119)	0.007±0.046	0.015 (−0.174, 0.202)	t_110.6_ = 0.15 (0.88)
Condition	100 (119)	−0.245±0.146	−0.161 (−0.335, 0.029)	t_112.6_ = −1.68 (0.10)
Age	38 (49)	0.190±0.159	1.825 (−1.19, 4.819)	t_43.9_ = 1.20 (0.24)
Heterophile:Lymphocyte	31 (31)	0.026±0.148	0.033 (−0.309, 0.364)	t_30_ = 0.18 (0.86)
Bactericidal assay	31 (31)	0.321±0.457	0.127 (−0.226, 0.437)	t_30_ = 0.70 (0.49)
Song rate during playback	50 (55)	0.089±0.041	0.290 (0.018, 0.504)	t_46.1_ = 2.15 (0.04)
Flights Across Speaker	50 (55)	0.160±0.070	0.309 (0.039, 0.519)	t_46.7_ = 2.30 (0.03)
Time Close to Speaker	50 (55)	0.801±0.338	0.326 (0.050, 0.537)	t_51_ = 2.37 (0.02)
**Polygyny**	**99 (118)**	**0.289±0.091**	**2.272 (0.835, 3.698)**	**t_97.9_ = 3.19 (0.001)**
**Maintaining WP success**	**85 (101)**	**1.237±0.300**	**0.464 (0.254, 0.616)**	**t_95.4_ = 4.12 (0.0001)**
EP success	99 (117)	0.170±0.075	0.845 (0.102, 1.584)	t_111.1_ = 2.26 (0.03)

Trill measures were the dependent variables, and reproductive success the independent variables, in mixed-effects models controlling for male identity, year, trill type, pitch, trill duration, and the time of the trill in the song. For male quality and mating success measures, reproductive success was the dependent variable, in mixed models controlling for year and male identity. For age, the effect estimate is the difference between second-year and after-second-year males, and the effect size is Cohen’s d. For continuous variables, effect estimates are the slope, and the effect size is partial r. Abbreviations: WP, within-pair. EP, extra-pair.

## Discussion

These results indicate that low vocal deviation and high trill consistency are not used as signals of high male quality in house wrens. Vocal deviation and trill consistency did appear to reflect underlying singing ability, since trill measures were repeatable and correlated with each other. However, these measures of trill quality mostly did not correlate with body condition or health (Table 2), and males with “better” songs did not have higher mating success (Tables 3, 4, 5, and 6) or higher reproductive success (Table 7). In three of the four analyses where mating success and song quality were significantly related, less successful males had better songs, the opposite of what I had predicted, and the effect sizes were small. I therefore conclude that there is little, if any, evidence that vocal deviation and trill consistency affect mating interactions in house wrens.

### Song and Male Quality

While most measures of male quality did not correlate with trill quality (also see [24]), older males did sing with higher trill consistency (Table 2), a result that is highly consistent with the current literature. In several other species (reviewed by [33], [76]), as in house wrens, older males sing more consistently than males in their first breeding season. Moreover, a longitudinal study in great tits (*Parus major*) showed that consistency decreased with age among relatively old males [37], a pattern also present in house wrens (though with limited sample size). Relatively young males are thought to improve their song consistency over time as they practice singing, generating the prediction that males with higher song output should also sing relatively consistently (reviewed in [33]). Supporting this prediction, house wrens with more consistent trills sang at higher rates in response to playback. If song rate during playback reflects a male’s overall song rate, perhaps these males have simply practiced their songs more and therefore have higher trill consistency. Thus, trill consistency might honestly indicate male age, or the extent to which he has practiced singing, in house wrens.

However, older males did not have higher mating or reproductive success, suggesting that a signal of age may not be useful in this population. Older males did not have higher WP or EP success, were not more likely to become polygynous, and did not fledge more offspring. Sample sizes for age comparisons were somewhat limited.

Trill consistency also correlated positively with another aspect of playback response, the amount of time spent close to the speaker. This result is surprising, since the trill consistency of a playback stimulus does not affect how house wren males respond to playback [30]. However, the effect size is small, suggesting that the statistical significance may be simply due to the high power because of the large number of trills measured.

### Song Quality Relative to Mating and Reproductive Success

Males that lost a higher proportion of WP paternity sang “better” trills (lower vocal deviation and higher trill consistency), and males that gained EP success in other nests sang with “worse” vocal deviation than males that failed to gain EP offspring elsewhere. These patterns are in the opposite direction from the predictions. It is plausible that investment in song trades off against investment in an unmeasured aspect of male quality that confers higher mating success, although in this case, the benefit of investing in song, which apparently gives little or no reproductive advantage, is unclear. Males that gained EP success did have higher trill consistency than males that did not, a relationship in the predicted direction, and this relationship may contribute to the trend for males with higher trill consistency to have higher reproductive success (Table 7). However, effect sizes for all of these relationships were low. Moreover, paired comparisons of EP males to the WP males they cuckolded did not reveal the same patterns (Table 6). Because a paired test should be more powerful at detecting biologically relevant patterns of cuckoldry, I suspect that the significant differences between males with and without EP success, and the correlation between trill measures and the proportion of social offspring sired, are also caused by the large number of trills measured.

Differences in mating success could be driven either by male-male competition or female choice. If male-male competition has the stronger effect on mating success, the lack of a strong effect of song on mating success is not surprising: vocal deviation and trill consistency do not appear to play an important role in male-male interactions in house wrens [30]. If female choice has the stronger effect on mating success, it appears that females do not base their choices on vocal deviation or trill consistency, since these trill measures do not relate to mating success. Alternatively, females may assess males based on spontaneous song but not on songs sung during territorial conflicts; since this study relied primarily on songs recorded during and immediately after playback, such an effect would not have been detected. However, house wrens do not have qualitatively different singing styles during playback and spontaneous singing, and moreover, females may be better able to compare two males’ singing ability when those males are countersinging [77], which would suggest that songs recorded during playback should be particularly relevant to females. A direct test of female preferences was not possible in this species.

It is thought that birds perceive frequency ratios rather than frequency bandwidths [78], [79], which suggests that it would be more biologically relevant to calculate vocal deviation based on frequency ratio rather than the standard measurement, frequency bandwidth (B. Lohr, pers. comm.; also see [30]). I investigated a ratio-based measure and how it related to male quality, mating success, and reproductive success [80]. However, the ratio-based and bandwidth-based measures of vocal deviation showed similar patterns, and the bandwidth-based measure had, if anything, stronger relationships with male quality and success measures [80].

The hypothesis that sexual signals are pivotal in EP mating decisions is commonly cited, but not supported in a recent meta-analysis of EP paternity in birds [81]. Similarly, a recent, thorough study of song sparrows (*Melospiza melodia*), a species where a great deal is understood about how song functions in male-male communication, found no differences in song between EP and WP males [82]. Perhaps researchers have not yet determined which aspects of signals are the salient ones for EP mating, or perhaps EP mating in many species is not driven by differences in male quality or signaling ability.

### Male Quality and Mating Success Relative to Reproductive Success

Polygynous males and males that maintained a higher proportion of WP paternity, unsurprisingly, had higher annual reproductive success than monogamous males and males with a lower proportion of WP paternity (Table 7). Males that gained some EP success showed a strong tendency to have higher reproductive success. This result is consistent with the finding that polygyny has a stronger effect on variation in male reproductive success than EP paternity does [48]. In contrast to other previous studies, polygynous males in this study did not lose more WP paternity in their secondary nests than in their primary nests (c.f., [65]). As a further contrast with previous work, I found no relationship between age and mating success, while Soukup and Thompson [65] found that older male house wrens tend to be less likely to be cuckolded and are significantly more likely to be polygynous than younger males. There may be intraspecific variation in EP mating behaviors (e.g., [83]).

Other measures of male quality did not relate to mating or reproductive success, though there were intriguing trends for more aggressive males to have higher reproductive success. Previous work in house wrens shows that EP offspring are not healthier than their WP half-siblings [84], so it is perhaps unsurprising that health measures were not related to EP or WP success. Moreover, body condition generally does not differ between EP and WP males across many species of birds [81], suggesting that the typical measures of body condition are not meaningful in birds, or that body condition is not relevant to EP mating decisions.

While much of the work on house wrens has focused on life history rather than sexual selection, it is interesting to note that we still do not know what male traits confer a mating and reproductive success advantage in this intensively-studied species. Nests initiated late in the season may be more likely to contain EP offspring [85] (though not in this population [53]), but this effect appears to be independent of the quality of the male himself [85]. Rare alleles at some loci [86] correlate with EP success, but that pattern was not observed in the same individuals when additional loci were considered [87]. As described in the introduction, male song appears to play a role in attracting females, but the nature of that role, and what aspects of a song make it particularly attractive, is still unclear [45]–[47]. Post-copulatory processes could, theoretically, also play a role in creating variation in male EP and WP success, but no correlations have been found between success in EP paternity and sperm characteristics [80]. The dynamics of mate choice in this species remain a mystery.

### Conclusions

Physically challenging aspects of song production may be likely to be honest signals of male quality [6]. However, sexual selection can promote different signal properties in different lineages [8], [88], and neither vocal deviation or trill consistency appears to be under sexual selection in house wrens. Perhaps complexities of house wren song structure complicate the interpretation of these particular parameters for listening birds (as I argue in [30]), and another song parameter, such as complexity itself, could be the target for sexual selection in house wrens.
